# Lactoferrin and lysozyme to reduce environmental enteric dysfunction and stunting in Malawian children: study protocol for a randomized controlled trial

**DOI:** 10.1186/s13063-017-2278-8

**Published:** 2017-11-06

**Authors:** William D. Cheng, Karl J. Wold, Nicole S. Benzoni, Chrissie Thakwalakwa, Kenneth M. Maleta, Mark J. Manary, Indi Trehan

**Affiliations:** 1 Department of Pediatrics, Washington University in St. Louis, One Children’s Place, Campus Box 8116, Saint Louis, MO 63110 USA; 2 School of Public Health and Family Medicine, University of Malawi, Blantyre, Malawi; 3 Children’s Nutrition Research Center, Baylor College of Medicine, Houston, TX USA; 4 Department of Paediatrics and Child Health, University of Malawi, Blantyre, Malawi; 5 Lao Friends Hospital for Children, Luang Prabang, Lao PDR

**Keywords:** Environmental enteric dysfunction, Environmental enteropathy, Inflammation, Lactoferrin, Lactulose, Lysozyme, Malnutrition, Stunting

## Abstract

**Background:**

Chronic childhood malnutrition, as manifested by stunted linear growth, remains a persistent barrier to optimal child growth and societal development. Environmental enteric dysfunction (EED) is a significant underlying factor in the causal pathway to stunting, delayed cognitive development, and ultimately morbidity and mortality. Effective therapies against EED and stunting are lacking and further clinical trials are warranted to effectively identify and operationalize interventions.

**Methods/design:**

A prospective randomized placebo-controlled parallel-group randomized controlled trial will be conducted to determine if a daily supplement of lactoferrin and lysozyme, two important proteins found in breast milk, can decrease the burden of EED and stunting in rural Malawian children aged 12–23 months old. The intervention and control groups will have a sample size of 86 subjects each. All field and laboratory researchers will be blinded to the assigned intervention group, as will the subjects and their caregivers. The percentage of ingested lactulose excreted in the urine (Δ%L) after 4 h will be used as the biomarker for EED and linear growth as the measure of chronic malnutrition (stunting). The primary outcomes of interest will be change in Δ%L from baseline to 8 weeks and to 16 weeks. Intention-to-treat analyses will be used.

**Discussion:**

A rigorous clinical trial design will be used to assess the biologically plausible use of lactoferrin and lysozyme as dietary supplements for children at high risk for EED. If proven effective, these safe proteins may serve to markedly reduce the burden of childhood malnutrition and improve survival.

**Trial Registration:**

Clinicaltrials.gov, NCT02925026. Registered on 4 October 2016.

**Electronic supplementary material:**

The online version of this article (doi:10.1186/s13063-017-2278-8) contains supplementary material, which is available to authorized users.

## Background

Undernutrition is directly or indirectly implicated in 45% of all deaths worldwide among children less than 5 years of age. Linear growth stunting is a common manifestation of prenatal and chronic early childhood undernutrition, afflicting at least 156 million children worldwide below 5 years of age. Stunting serves as a clinical marker for lifelong impairments in physical, immunological, neurocognitive, and socioeconomic potential. Although the prevalence of stunting is decreasing globally, it remains a significant problem and interventions to ameliorate stunting will be needed if the global community is to achieve the Sustainable Development Goals related to childhood health and nutrition [1]. Most of the irreversible stunting suffered by children in resource-limited settings occurs within the first 1000 days after conception, and is linked to pre-conception maternal nutritional status, nutrition and infections during pregnancy, postnatal nutrition, and gut health during this time [2]. Gut health, as defined here, is affected by nutrient intake and infectious assaults on the gut, and the inflammatory balance the gut must strike in optimizing the first while ameliorating the latter. Interventions to optimize gut health during early childhood are urgently needed in order to decrease the risk of stunting and subsequent morbidity and mortality. For the study presented here, we take the approach of improving the nutritional composition of complementary foods by supplementation with two compounds, lactoferrin and lysozyme, found in breast milk.

### Environmental enteric dysfunction

Environmental enteric dysfunction (EED) is a subclinical chronic inflammatory condition of the gut. Known in the past as tropical sprue, tropical enteropathy, or environmental enteropathy, it is condition that primarily affects rural, poor, but seemingly healthy children, most prominently below 2–3 years of age [3–5]. EED is histologically characterized by diffuse small bowel villous atrophy and T-cell infiltration of the intestinal mucosa that causes chronic immunostimulation [6], leading to increased intestinal permeability and impaired mucosal barrier function, which further leads to translocation of gut microbes and endotoxins, and nutrient malabsorption [7]. Although EED itself is a subclinical condition, affected children are subjected to increased rates of infection, poorer response to oral vaccines, and growth stunting.

Interventions aimed at combating EED have had mixed results. Trials involving antibiotics [8] and probiotics [9] to modulate the gut microbiota and decrease microbial-stimulated inflammatory responses have had limited success. Supplementation with multiple vitamin A [10], alanyl-glutamine [11], long-chain polyunsaturated fatty acids [12], albendazole [13], zinc [13], multiple micronutrient supplements [14], and mesalazine [15] have all been shown to at least transiently benefit small intestine function. However, these benefits have not always been consistent—even in combination—among children in the periods of highest food insecurity [16], and any demonstrated improvements in linear growth and development remain lacking. Given that malnutrition to this day still accounts for a significant amount of morbidity and mortality in children less than 5 years of age, interventions aimed at reducing EED are integral to improving child survival worldwide [17].

### Lactoferrin and lysozyme

Breastfeeding provides innumerable benefits to infants and children, both in the short and long term. Infants in low- and middle-income countries who have never breastfed are 14 times likely to die from all causes compared to exclusively breastfed infants in the first 5 months of life [18]; most of this benefit stems from a lower risk of acute infections.

Similar to children with minimal dietary diversity, breast milk has also been shown to be protective against increased intestinal permeability [19]. Lactoferrin and lysozyme are two proteins present in substantial concentrations within various human mucosal secretions, particularly breast milk [20]. Lactoferrin is an 80-kDa iron-binding glycoprotein which accounts for 25% of the protein in breast milk (1–3 g/mL) and functions to sequester iron, thereby inhibiting bacterial growth [19, 21]. Lysozyme is a 15-kDa protein present in breast milk at 0.1–0.3 mg/mL that acts as an antimicrobial by lysing a specific connection within the peptidoglycan layer of bacterial cell walls [22].

Previous research has demonstrated that both lactoferrin and lysozyme supplementation are safe and efficacious in children as young as 5 months of age [23]. Lactoferrin has been shown to improve hematocrit levels and decrease the rate of lower respiratory tract infections in neonates without adverse effects [24]. Lysozyme levels in breast milk have been positively correlated with neonatal weight gain [25].

Lysozyme has also been shown to modulate inflammatory response and gut structure, as well as alter microbiota in animal models. One study showed that pigs which were fed human lysozyme-enriched milk expressed significantly elevated blood levels of the anti-inflammatory cytokine transforming growth factor (TGF)-β1 and tended to have longer villi with thinner lamina propia, which are linked to increased intestinal surface area and increased nutritional absorptive capacity [26]. In this animal model, lysozyme also led to altered gut microbiota, relatively enriched in *Bifidobacteriacea* and *Lactobacillacea*, two populations associated with a healthy microbiome, and decreased *Mycobacteriaceae* and *Campylobacterales* populations, which are associated with disease [27, 28]. Another study of supplementation with transgenic milk containing human lactoferrin in malnourished pigs showed benefits to jejunal architecture, reduced intestinal permeability, and promotion of weight gain [29].

In combination, lactoferrin and lysozyme have been shown to work synergistically as bactericidal agents against both Gram-negative and Gram-positive bacteria. Lactoferrin destabilizes the outer membrane likely through a mechanism involving iron sequestration and binding to membrane lipopolysaccharide, while lysozyme acts on the inner peptidoglycan membrane [22, 30, 31]. Importantly, both are resistant to proteolytic degradation [32, 33], which is essential for any therapeutic use as an oral agent in humans.

In a study of Peruvian children between the ages of 5 and 33 months, supplementing oral rehydration solutions with lactoferrin and lysozyme in the setting of acute diarrhea with dehydration decreased the severity and duration of diarrheal episodes, without any adverse effects related to treatment [34]. These therapeutic properties make lactoferrin and lysozyme attractive candidates for the prevention and treatment of EED, and more broadly may be useful supplements for infants who cannot breastfeed or who are weaning from breast milk but may still benefit from their therapeutic properties.

### Study goals

There are numerous factors that contribute to stunting among impoverished children worldwide, poor gut health being among the most important. We plan to investigate the effect of nutritional supplementation with lactoferrin and lysozyme on the growth and gut health of children in rural Malawi, a setting where over 80% of children have EED [8, 9, 13, 14, 16, 35, 36].

## Methods/design

### Study design

**Fig. 1:**
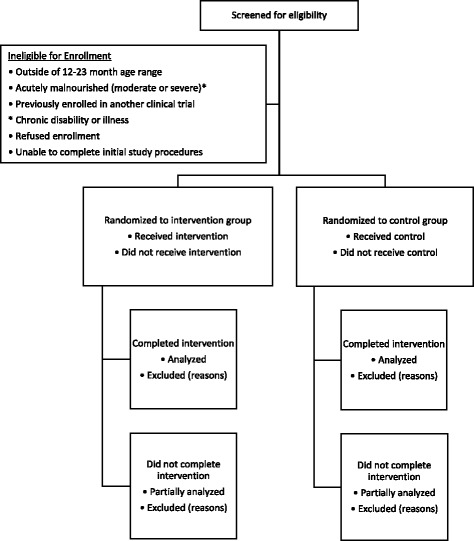
Anticipated patient flow for the randomized clinical trial. *Defined as having weight-for-height *Z* score (WHZ) < –2, mid-upper arm circumference (MUAC) < 12.5 cm, and/or bilateral pitting edema suggestive of kwashiorkor

This will be a prospective, randomized, double-masked controlled clinical trial to assess the effect of lactoferrin and lysozyme supplementation on growth and gut health in rural Malawian children. Children between the ages of 12 and 23 months will be eligible for enrollment, and those enrolled will receive the intervention daily for 16 weeks. A 1:1 allocation ratio will be used to assign children to either the intervention or placebo groups. The anticipated trial flow is depicted in Fig. 1. The Standard Protocol Items, Recommendations for Interventional Trials (SPIRIT) checklist is available as Additional file 1.

### Study setting

This study will enroll children in the villages surrounding the community of Mmwenye, Machinga District, Malawi. Most villagers work as subsistence farmers with maize as the primary crop. Water is primarily acquired from boreholes or carried by hand from nearby streams. Homes are generally small, with foundations constructed of mud brick and roofs of thatch or sheet metal. It is not uncommon for multiple generations to live within the same house. Defecation and urination are primarily done in the open [37] or for some families in pit latrines dug into the ground. Children interact frequently with farm animals including chickens, goat, and cattle, some of which are permitted within houses. Geophagy is common [38, 39]. Electricity, running water, and other utilities are generally absent; most cooking is accomplished on open wood or charcoal fires and most cleaning is performed at streams or with water obtained from those streams. Most household responsibilities and child-rearing are performed by women. It is common for women to marry and assume household responsibilities relatively early in life, and thus most women are illiterate and have not completed primary school. Most hospitals are situated in the larger cities and are therefore prohibitively far away from villagers; more commonly, local health clinics have only modest resources and staffing. Health promotion and disease prevention is primarily accomplished in the community by Health Surveillance Assistants (HSAs) [40].

### Ethical considerations

The study has been structured in accordance with the Helsinki Declaration [41] and approved by the University of Malawi College of Medicine Research and Ethics Committee and Washington University School of Medicine Human Research Protection Office. Consent for conducting the study was obtained from district health officials and community leaders. Prior to enrollment, large-group community meetings will be held to inform potential participants, their caregivers, and any interested members of the community about the study and answer their concerns. Consent from all caregivers will be obtained in written and verbal forms by native Chichewa-speaking Malawian research nurses, and those who cannot sign their names will submit thumbprints instead (Additional file 2).

Given that the intervention proteins are a normal component of breast milk, it is extremely unlikely that any adverse effects will be observed. No specific data safety monitoring committee will be used, but rather daily monitoring for any adverse events will be conducted by the field staff and reviewed and investigated by the study principal investigators. Additionally, at each follow-up visit caretakers will be specifically queried about the presence of any ill symptoms or unusual clinical events in their children in an attempt to proactively identify any untoward effects.

### Eligibility criteria

All children within the enrollment ages of 12 to 23 months of age who live within walking distance of the Mmwenye village clinic site will be screened for eligibility. Participants will be excluded if the child cannot drink 20 mL of sugar water or otherwise give the necessary biological samples, or if the family plans to move from the area during the study duration. Children will also be excluded if they are moderately or severely malnourished (defined as having a weight-for-height *Z* score < –2, mid-upper arm circumference (MUAC) < 12.5 cm, or presence of pitting edema), have congenital disorders or chronic diseases, dairy allergy, or have acute illnesses such as fever, cough, or diarrhea.

### Outcomes and sample size

The primary outcomes of interest will be improvement of a major biomarker of EED, the percent of ingested lactulose excreted in the urine (Δ%L), from baseline to 8 weeks and to 16 weeks. Linear growth has been shown to best correlate with this component of the traditional dual-sugar absorption test [42]. The enrollment goal will be at least 86 subjects in each of two equally sized intervention or control groups. This sample size was chosen to detect a decrease of 0.05% in Δ%L between the two groups (considered to be a clinically significant improvement in gut barrier function) with an alpha value of 5% and 90% power. This assumes that urinary lactulose measurements will be distributed similarly to prior studies that showed that more than 80% of apparently healthy rural Malawian children had EED. Additional children will be enrolled to account for children who drop out or are excluded during the course of the study, as well as for some children who will not have a satisfactory data collection due to technical issues with the urine collection. The differences in lactulose recovery before and after the intervention will be calculated and compared between the intervention and control groups using *t* tests if the data are normally distributed or the non-parametric Wilcoxon Rank Sum Test if the data are not normally distributed.

Secondary outcomes of interest include linear and ponderal growth, adverse gastrointestinal symptoms, and any side effects due to the interventions. Linear growth will be calculated as the change in length over time, expressed as mm/day. Weight change will be calculated as the change in weight over time, normalized to the enrollment weight, and expressed as g/kg/day. Exploratory analyses of mRNA markers of intestinal inflammation found in the stool will also be conducted as part of our ongoing investigations into identifying novel biomarkers for EED [43, 44].

### Intervention groups

Following randomization, the experimental group will receive 1.5 g of bovine lactoferrin powder and 40 g of unpurified rice powder that contains 0.2 g of recombinant human lysozyme daily. The control group will receive 41.5 g of locally sourced rice powder daily. The amount of enzymes to be consumed per child per day is a conservative estimate based on the amount a typical breastfeeding 12-month old would receive daily and is consistent with previous studies [34, 45, 46]. Each group will receive their respective interventions for 16 weeks.

Bovine lactoferrin has been granted Generally Regarded as Safe (GRAS) status by the US Food and Drug Administration, and is widely used in studies of lactoferrin efficacy [23, 47, 48]. It will be acquired from FrieslandCampina (Amersfoort, Netherlands).

Bovine milk contains only trace amounts of lysozyme and is therefore not a viable source for this enzyme, but genetic modification has successfully integrated recombinant human lysozyme into rice. The transgenic rice has a normal plant phenotype; after harvest, the recombinant proteins are extracted and purified from milled rice powder as partially iron-saturated lysozyme [34]. The amino acid profile, iron content, peptide matching, chemical characterization, and functionality is identical to human lysozyme present in breast milk [49]. Ventria (Junction City, Kansas, USA) will be the supplier of the rice-based lysozyme product used in this study [34].

For the control group, a local variety of rice called faya will be used. It is an aromatic rice locally grown in Malawi with a nutritional profile similar to that used in the experimental formulation. The only processing this rice will undergo will be mechanical grinding; there will be no cooking, addition of chemicals, or other processes.

### Preparation of lactoferrin and lysozyme

Participants will receive their interventions packaged in clear plastic jars with adequate room for shaking and mixing, and will be instructed to store their interventions in a cool location with the caps screwed on tightly. Caregivers will be instructed to add the interventions to the child’s “phala”, which is the traditional morning dish of maize porridge that is commonly fed as the preferred complementary food. Phala is made by adding maize flour to boiling water and stirring over heat for about 15 min until thickened to the desired consistency. To avoid any denaturation or alteration of the interventions that may come with boiling, caregivers will be instructed to add the interventions after cooking is complete. Each participant will be given a small scoop with which to portion the correct amount of intervention, and they will be taught to mix the specified number of these scoops into the cooked phala. Acceptability tests in local children have shown that both the experimental and control interventions are palatable when mixed into this phala, with no adverse reactions noted.

### Randomization and blinding

Each number from 1 to 250 will be randomly pr-assigned to either the control or experimental group by a random number generator, with 125 numbers assigned to each group. These numbers will be assigned in sequential order to participants as they are enrolled. The staff that generate the randomization list, and prepare and label each intervention/control jar with study numbers will do so prior to delivery of the interventions to the field and those staff will have no further involvement in the study. A designated field team member not involved in sample collection or anthropometric measurements will distribute the jars to the study participants. These field researchers will be trained in anthropometry, study protocols, the assessment of acute malnutrition, and good clinical research practices by the senior physicians and investigators. Researchers involved in the collection of samples and analysis of data will not directly handle, distribute, or view the intervention flours, nor will any of the paperwork they handle include information about the assigned intervention group. Care will be taken to ensure that the experimental and control powders look as similar as possible, but there are very subtle color, particulate, and texture differences between the two. Although caregivers will not be told to which group their children are randomized, it will be possible for them to differentiate the two different powders being used in the study should they undertake a very close comparison with another subject’s flour.

### Study participation

On the enrollment date, children will be screened for eligibility via clinical anthropometric measurements and a short questionnaire regarding recent illnesses. Children without any exclusion criteria will be given a study number, and will complete the lactulose absorption test, stool collection, and blood draw after consent is provided. HSAs will work with caregivers to complete a questionnaire that includes basic sociodemographic, economic, sanitary, and dietary data specific to lactoferrin- and lysozyme-containing foods, along with an account of recent illnesses in the previous 7 days. After completion of sample collection, each participant will receive a 4-week supply of the intervention corresponding to their assigned study number. Local staff will then educate caregivers on how to prepare and feed the interventions to the children. Recruitment of trial participants began in October 2016 and the trial is ongoing.

**Fig. 2:**
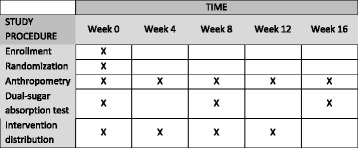
SPIRIT figure: study participation schedule for subjects

At subsequent monthly visits, study participants will return for anthropometric measurements, with or without sample collections. Questionnaires that primarily detail recent illnesses, medical visits, diet, and compliance by asking the caretaker how many days of the intervention were missed will be completed. The complete schedule of study visits, testing, and distribution of interventions is provided in the SPIRIT format in Fig. 2.

### Anthropometry

Anthropometric data will be obtained at every visit. Weight will be assessed to the nearest 5 g using standard digital scales (Seca 334, Chino, California, USA) and height will be measured to the nearest 0.1 cm using standard rigid height boards (Seca 417, Chino, California, USA). MUAC will be measured to the nearest 0.1 cm using standard flexible tape inserts (TALC, St. Albans, UK). Each child will be assessed for edema indicative of kwashiorkor by pressing on the dorsum of the left foot. The field research staff supervising anthropometry have been working in similar field sites and study conditions and trials for 5–15 years each, and will ensure that any junior research staff are fully trained and vetted prior to initiating the study. A random sampling of measurements each day will also be conducted by at least two field staff in order to verify that consistent and accurate measurements are being taken and corrective measures initiated as soon as any inaccuracies are identified.

Anthropometric *Z* scores will be calculated based on the 2006 World Health Organization (WHO) Multicentre Growth Reference Survey [50]. During both enrollment and subsequent visits, any child with a weight-for-height *Z* score less than –2, MUAC less than 12.5 cm, or presence of pitting edema will be classified as acutely malnourished and excluded from the study. Children excluded due to acute malnutrition will then be enrolled in supplementary or therapeutic feeding programs and receive usual medical care according to standard protocols [51–53].

### Lactulose absorption test

The lactulose absorption test is a modification of the dual-sugar absorption test [54] and will be used as a measure of gut integrity. This test has previously been correlated with histological changes in the small bowel [55], severity of diarrhea [56], micronutrient status [57, 58], metabolic abnormalities [59], and anthropometric growth [60]. This test involves the oral ingestion of a sugar solution with subsequent urine collection to determine the degree to which the sugars are absorbed within the intestine and enter the systemic circulation.

Lactulose is a large disaccharide that cannot cross these tight junctions, and can only traverse the intestinal epithelium in large quantities if there are significant disruptions [61]. The percentage of ingested lactulose excreted in the urine is a measure of intestinal mucosal permeability, with a higher percentage signifying increased intestinal permeability.

Children will be asked to take no food (water and breast milk are permitted) after 10 pm on the evening before each sample collection date (weeks 0, 8, and 16). Each child will meet at the community meeting point in the village at 6 am with the research team. Each child then will drink 20 mL of a solution containing 5 g lactulose. Children who spit up or vomit the solution will return on a subsequent day to try again. Adhesive urine bags will then be placed on the child’s perineum to capture each urination. Once a child has voided any quantity of urine, the bag will be removed and the urine will be transferred to a clean container specific to the child, and then a new urine bag will be placed on the child. Thimerosol (10 mg) dissolved in 20 μL of water will be added to each child’s urine container to prevent bacterial degradation of the sugars. Caretakers and children are told to avoid breastfeeding, and will remain at the community meeting point for the next 4 h. Four hours after drinking the sugar solution, children will be encouraged to void and complete the urine collection, after which the total volume of urine will be quantified and aliquots portioned into cryovials. These samples will be stored in liquid nitrogen tanks in the field and then stored in –80 °C freezers until they can be transported back to the United States. The urinary concentration of lactulose will be measured by high-pressure liquid chromatography (HPLC) as described previously [62]. The total excretion of lactulose will be determined by multiplying this concentration by the total volume of urine.

### Data management and analysis

Clinical data focusing on demographic and anthropometric values will be collected on standardized forms by field workers. Data will be double-entered into a password-protected Microsoft Access database by research assistants blinded to the intervention assignment. All discrepancies will be resolved by examination of the original forms and discussion with the relevant field workers. After entering all data and resolving inconsistencies, the data set will be locked. For anthropometric values, comparisons will be made between the lactoferrin-lysozyme mixture and the control rice flour groups. Fisher’s exact test will generally be used to compare discrete parameters, and Student’s *t* test will be used for continuous parameters. A difference with *P* < 0.05 will be considered statistically significant. These statistical methods will also be used for all clinical data and EED measures.

All trial data, including personal information, will be kept in password-protected encrypted computer databases on password-protected computers. Paper records will be kept in locked file cabinets and the research team’s headquarters in locked buildings. Given the relatively short duration of the study and the need to analyze all urine concentrations simultaneously at the end of the study, no interim analyses are planned and no stopping guidelines have been established. The full protocol, participant-level dataset, and details of all statistical analyses will be made available to any interested parties after the conclusion of the trial by contacting the principal investigators.

## Discussion

Malnutrition and chronic enteric infections are closely intertwined global health problems. Ever since the 1960s, it has been known that the two in concert cause long-term growth stunting and impaired cognitive development [63]. Since then, studies have quantified the effect of enteric infections and have found that diarrheal episodes in the first 2 years of life can account for 25% or more of stunting [64], and newer research suggests a link to non-communicable diseases such as cardiovascular disease and type II diabetes [65]. Central to the treatment of stunting will be interventions targeted against EED. Past studies have tested pharmacologic interventions against EED, but effects have been minimal [66].

Breast milk provides a number of benefits for developing infants, including the provision of energy, gut maturation, and immunologic functions [67]. Though the use breastfeeding as an intervention to treat undernutrition has shown only modest reduction in stunting, it decreases mortality rates significantly [2]. Thus, use of breast milk components in supplements may be useful in the prevention and/or treatment of EED. Lactoferrin and lysozyme are of particular interest as they are both present in substantial concentrations within breast milk, and increase substantially in concentration during lactation [68]. Their antimicrobial properties have been demonstrated in vitro [22] and in vivo [34], along with potential benefits to intestinal health [34], and it is our hope that their use as interventions may help attenuate EED.

One limitation of this study is the lack of complete blinding. The control and experimental interventions have physical differences that may be noticeable to discerning caregivers, who may subsequently exchange their interventions with others. We believe that this will be an unlikely occurrence since past research in this area of Malawi did not demonstrate any evidence of sharing or trading for food [69] or pharmacological [70] interventions. Extensive community education of local officials, caregivers, and workers will stress the importance of complying with the assigned interventions, which should decrease the occurrence of sharing. Preliminary acceptability studies showed that children liked the interventions equally, which should also decrease the motivation for trading. There is also potential for sharing of the interventions with other family members, but this is relatively unlikely as the amount administered is unlikely to be satisfying to hungry family members.

## Trial status

Recruitment and follow-up of trial participants is ongoing.

## Additional files

Additional file 1: SPIRIT 2013 checklist: recommended items to address in a clinical trial protocol and related documents. (DOC 120 kb)

Additional file 2: Assessment of the change in environmental enteropathy after supplementation with bovine lactoferrin and recombinant human lysozyme. (PDF 1177 kb)

## Abbreviations

EED: Environmental enteric dysfunction
GRAS: Generally Regarded as Safe
HPLC: High-pressure liquid chromatography
HSA: Health Surveillance Assistant
MUAC: Mid-upper arm circumference
SPIRIT: Standard Protocol Items, Recommendations for Interventional Trials
WHO: World Health Organization

## References

[1] World Health Organization (2016). World health statistics 2016.

[2] Bhutta ZA, Das JK, Rizvi A, Gaffey MF, Walker N, Horton S (2013). Evidence-based interventions for improvement of maternal and child nutrition: what can be done and at what cost?. Lancet.

[3] Crane RJ, Jones KD, Berkley JA (2015). Environmental enteric dysfunction: an overview. Food Nutr Bull.

[4] Trehan I, Kelly P, Shaikh N, Manary MJ (2016). New insights into environmental enteric dysfunction. Arch Dis Child.

[5] Watanabe K, Petri WA (2016). Environmental enteropathy: elusive but significant subclinical abnormalities in developing countries. EBioMedicine.

[6] Keusch GT, Denno DM, Black RE, Duggan C, Guerrant RL, Lavery JV (2014). Environmental enteric dysfunction: pathogenesis, diagnosis, and clinical consequences. Clin Infect Dis.

[7] Kelly P, Besa E, Zyambo K, Louis-Auguste J, Lees J, Banda T (2016). Endomicroscopic and transcriptomic analysis of impaired barrier function and malabsorption in environmental enteropathy. PLoS Negl Trop Dis.

[8] Trehan I, Shulman RJ, Ou CN, Maleta K, Manary MJ (2009). A randomized, double-blind, placebo-controlled trial of rifaximin, a nonabsorbable antibiotic, in the treatment of tropical enteropathy. Am J Gastroenterol.

[9] Galpin L, Manary MJ, Fleming K, Ou CN, Ashorn P, Shulman RJ (2005). Effect of Lactobacillus GG on intestinal integrity in Malawian children at risk of tropical enteropathy. Am J Clin Nutr.

[10] Thurnham DI, Northrop-Clewes CA, McCullough FS, Das BS, Lunn PG (2000). Innate immunity, gut integrity, and vitamin a in Gambian and Indian infants. J Infect Dis.

[11] Lima NL, Soares AM, Mota RM, Monteiro HS, Guerrant RL, Lima AA (2007). Wasting and intestinal barrier function in children taking alanyl-glutamine-supplemented enteral formula. J Pediatr Gastroenterol Nutr.

[12] van der Merwe LF, Moore SE, Fulford AJ, Halliday KE, Drammeh S, Young S (2013). Long-chain pufa supplementation in rural African infants: a randomized controlled trial of effects on gut integrity, growth, and cognitive development. Am J Clin Nutr.

[13] Ryan KN, Stephenson KB, Trehan I, Shulman RJ, Thakwalakwa C, Murray E (2014). Zinc or albendazole attenuates the progression of environmental enteropathy: a randomized controlled trial. Clin Gastroenterol Hepatol.

[14] Smith HE, Ryan KN, Stephenson KB, Westcott C, Thakwalakwa C, Maleta K (2014). Multiple micronutrient supplementation transiently ameliorates environmental enteropathy in Malawian children aged 12–35 months in a randomized controlled clinical trial. J Nutr.

[15] Jones KD, Hunten-Kirsch B, Laving AM, Munyi CW, Ngari M, Mikusa J (2014). Mesalazine in the initial management of severely acutely malnourished children with environmental enteric dysfunction: a pilot randomized controlled trial. BMC Med.

[16] Wang AZ, Shulman RJ, Crocker AH, Thakwalakwa C, Maleta KM, Devaraj S (2017). A combined intervention of zinc, multiple micronutrients, and albendazole does not ameliorate environmental enteric dysfunction or stunting in rural Malawian children in a double-blind randomized controlled trial. J Nutr.

[17] Owino V, Ahmed T, Freemark M, Kelly P, Loy A, Manary M (2016). Environmental enteric dysfunction and growth failure/stunting in global child health. Pediatrics.

[18] Sankar MJ, Sinha B, Chowdhury R, Bhandari N, Taneja S, Martines J (2015). Optimal breastfeeding practices and infant and child mortality: a systematic review and meta-analysis. Acta Paediatr.

[19] Taylor SN, Basile LA, Ebeling M, Wagner CL (2009). Intestinal permeability in preterm infants by feeding type: mother’s milk versus formula. Breastfeed Med.

[20] Lönnerdal B (1985). Biochemistry and physiological function of human milk proteins. Am J Clin Nutr.

[21] Lönnerdal B, Iyer S (1995). Lactoferrin: molecular structure and biological function. Annu Rev Nutr.

[22] Ellison RT, Giehl TJ (1991). Killing of Gram-negative bacteria by lactoferrin and lysozyme. J Clin Invest.

[23] Ochoa TJ, Pezo A, Cruz K, Chea-Woo E, Cleary TG (2012). Clinical studies of lactoferrin in children. Biochem Cell Biol.

[24] King JC, Cummings GE, Guo N, Trivedi L, Readmond BX, Keane V (2007). A double-blind, placebo-controlled, pilot study of bovine lactoferrin supplementation in bottle-fed infants. J Pediatr Gastroenterol Nutr.

[25] Braun OH, Sandkühler H (1985). Relationships between lysozyme concentration of human milk, bacteriologic content, and weight gain of premature infants. J Pediatr Gastroenterol Nutr.

[26] Cooper CA, Brundige DR, Reh WA, Maga EA, Murray JD (2011). Lysozyme transgenic goats’ milk positively impacts intestinal cytokine expression and morphology. Transgenic Res.

[27] Ventura M, O'Flaherty S, Claesson MJ, Turroni F, Klaenhammer TR, van Sinderen D (2009). Genome-scale analyses of health-promoting bacteria: probiogenomics. Nat Rev Micro.

[28] Maga EA, Desai PT, Weimer BC, Dao N, Kültz D, Murray JD (2012). Consumption of lysozyme-rich milk can alter microbial fecal populations. Appl Environ Microbiol.

[29] Garas LC, Feltrin C, Hamilton MK, Hagey JV, Murray JD, Bertolini LR (2016). Milk with and without lactoferrin can influence intestinal damage in a pig model of malnutrition. Food Funct.

[30] Ellison RT, Giehl TJ, LaForce FM (1988). Damage of the outer membrane of enteric Gram-negative bacteria by lactoferrin and transferrin. Infect Immun.

[31] Leitch EC, Willcox MDP (1998). Synergic antistaphylococcal properties of lactoferrin and lysozyme. J Med Microbiol.

[32] De Laureto PP, De Filippis V, Scaramella E, Zambonin M, Fontana A (1995). Limited proteolysis of lysozyme in trifluoroethanol. Eur J Biochem.

[33] Kuwata H, Yamauchi K, Teraguchi S, Ushida Y, Shimokawa Y, Toida T (2001). Functional fragments of ingested lactoferrin are resistant to proteolytic degradation in the gastrointestinal tract of adult rats. J Nutr.

[34] Zavaleta N, Figueroa D, Rivera J, Sánchez J, Alfaro S, Lönnerdal B (2007). Efficacy of rice-based oral rehydration solution containing recombinant human lactoferrin and lysozyme in Peruvian children with acute diarrhea. J Pediatr Gastroenterol Nutr.

[35] Brewster DR, Manary MJ, Menzies IS, O'Loughlin EV, Henry RL (1997). Intestinal permeability in kwashiorkor. Arch Dis Child.

[36] Trehan I, Benzoni NS, Wang AZ, Bollinger LB, Ngoma TN, Chimimba UK (2015). Common beans and cowpeas as complementary foods to reduce environmental enteric dysfunction and stunting in Malawian children: study protocol for two randomized controlled trials. Trials.

[37] Spears D, Ghosh A, Cumming O (2013). Open defecation and childhood stunting in India: an ecological analysis of new data from 112 districts. PLoS One.

[38] George CM, Oldja L, Biswas S, Perin J, Lee GO, Kosek M (2015). Geophagy is associated with environmental enteropathy and stunting in children in rural Bangladesh. Am J Trop Med Hyg.

[39] Perin J, Thomas A, Oldja L, Ahmed S, Parvin T, Bhuyian SI (2016). Geophagy is associated with growth faltering in children in rural Bangladesh. J Pediatr.

[40] Zembe-Mkabile WZ, Jackson D, Sanders D, Besada D, Daniels K, Zamasiya T (2016). The ‘community’ in community case management of childhood illnesses in Malawi. Glob Health Action.

[41] World Medical Association (2013). World Medical Association Declaration of Helsinki: ethical principles for medical research involving human subjects. JAMA.

[42] Weisz AJ, Manary MJ, Stephenson K, Agapova S, Manary FG, Thakwalakwa C (2012). Abnormal gut integrity is associated with reduced linear growth in rural Malawian children. J Pediatr Gastroenterol Nutr.

[43] Ordiz MI, Shaikh N, Trehan I, Maleta K, Stauber J, Shulman R (2016). Environmental enteric dysfunction is associated with poor linear growth and can be identified by host fecal mRNAs. J Pediatr Gastroenterol Nutr.

[44] Yu J, Ordiz MI, Stauber J, Shaikh N, Trehan I, Barnell E (2016). Environmental enteric dysfunction includes a broad spectrum of inflammatory responses and epithelial repair processes. Cell Mol Gastroenterol Hepatol.

[45] Chandan RC, Shahani KM, Holly RG (1964). Lysozyme content of human milk. Nature.

[46] Ochoa TJ, Chea-Woo E, Baiocchi N, Pecho I, Campos M, Prada A (2013). Randomized double-blind controlled trial of bovine lactoferrin for prevention of diarrhea in children. J Pediatr.

[47] Food and Drug Administration. Agency Response Letter GRAS notice no. GRN 000465. http://www.fda.gov/Food/IngredientsPackagingLabeling/GRAS/NoticeInventory/ucm391549.htm. Accessed 2 Nov 2017.

[48] Atef Yekta M, Verdonck F, Van Den Broeck W, Goddeeris B, Cox E, Vanrompay D (2010). Lactoferrin inhibits e. Coli o157: H7 growth and attachment to intestinal epithelial cells. Vet Med.

[49] Huang J, Wu L, Yalda D, Adkins Y, Kelleher SL, Crane M (2002). Expression of functional recombinant human lysozyme in transgenic rice cell culture. Transgenic Res.

[50] WHO Multicentre Growth Reference Study Group (2006). Assessment of differences in linear growth among populations in the WHO Multicentre Growth Reference Study. Acta Paediatr Suppl.

[51] Trehan I, Manary MJ (2015). Management of severe acute malnutrition in low-income and middle-income countries. Arch Dis Child.

[52] World Health Organization (2012). Supplementary foods for the management of moderate acute malnutrition.

[53] World Health Organization (2013). Guideline: updates on the management of severe acute malnutrition in infants and children.

[54] Denno DM, VanBuskirk K, Nelson ZC, Musser CA, Hay Burgess DC, Tarr PI (2014). Use of the lactulose to mannitol ratio to evaluate childhood environmental enteric dysfunction: a systematic review. Clin Infect Dis.

[55] Ukabam SO, Cooper BT (1985). Small intestinal permeability as an indicator of jejunal mucosal recovery in patients with celiac sprue on a gluten-free diet. J Clin Gastroenterol.

[56] Zhang Y, Lee B, Thompson M, Glass R, Cama RI, Figueroa D (2000). Lactulose-mannitol intestinal permeability test in children with diarrhea caused by rotavirus and cryptosporidium. Diarrhea working group, Peru. J Pediatr Gastroenterol Nutr.

[57] Chen P, Soares AM, Lima AA, Gamble MV, Schorling JB, Conway M (2003). Association of vitamin a and zinc status with altered intestinal permeability: analyses of cohort data from northeastern Brazil. J Health Popul Nutr.

[58] Goto K, Chew F, Torun B, Peerson JM, Brown KH (1999). Epidemiology of altered intestinal permeability to lactulose and mannitol in Guatemalan infants. J Pediatr Gastroenterol Nutr.

[59] Semba RD, Shardell M, Trehan I, Moaddel R, Maleta KM, Ordiz MI (2016). Metabolic alterations in children with environmental enteric dysfunction. Sci Rep.

[60] Lunn PG, Northrop-Clewes CA, Downes RM (1991). Intestinal permeability, mucosal injury, and growth faltering in Gambian infants. Lancet.

[61] Mishra A, Makharia GK (2012). Techniques of functional and motility test: how to perform and interpret intestinal permeability. J Neurogastroenterol Motil.

[62] Shulman RJ, Eakin MN, Czyzewski DI, Jarrett M, Ou CN (2008). Increased gastrointestinal permeability and gut inflammation in children with functional abdominal pain and irritable bowel syndrome. J Pediatr.

[63] Scrimshaw NS, Taylor CE, Gordon JE (1968). Interactions of nutrition and infection. Monogr Ser World Health Organ.

[64] Checkley W, Buckley G, Gilman RH, Assis AM, Guerrant RL, Morris SS (2008). Multi-country analysis of the effects of diarrhoea on childhood stunting. Int J Epidemiol.

[65] Guerrant RL, DeBoer MD, Moore SR, Scharf RJ, Lima AA (2013). The impoverished gut—a triple burden of diarrhoea, stunting and chronic disease. Nat Rev Gastroenterol Hepatol.

[66] Petri WA, Naylor C, Haque R (2014). Environmental enteropathy and malnutrition: do we know enough to intervene?. BMC Med.

[67] Walker A (2010). Breast milk as the gold standard for protective nutrients. J Pediatr.

[68] Montagne P, Cuilliere ML, Mole C, Bene MC, Faure G (2001). Changes in lactoferrin and lysozyme levels in human milk during the first twelve weeks of lactation. Adv Exp Med Biol.

[69] Wang RJ, Trehan I, LaGrone LN, Weisz AJ, Thakwalakwa CM, Maleta KM (2013). Investigation of food acceptability and feeding practices for lipid nutrient supplements and blended flours used to treat moderate malnutrition. J Nutr Educ Behav.

[70] Trehan I, Goldbach HS, LaGrone LN, Meuli GJ, Wang RJ, Maleta KM (2013). Antibiotics as part of the management of severe acute malnutrition. N Engl J Med.

