# Supplementary material for: Lactoferrin and lysozyme to reduce environmental enteric dysfunction and stunting in Malawian children: study protocol for a randomized controlled trial
Source: Trials. 2017 Nov 6;18:523. doi: 10.1186/s13063-017-2278-8 (PMC5674751; doi:10.1186/s13063-017-2278-8)
Supplement: Supplementary file 2 — Assessment of the change in environmental enteropathy after supplementation with bovine lactoferrin and recombinant human lysozyme. (PDF 1177 kb) [file 13063_2017_2278_MOESM2_ESM.pdf]

Additional file 2

Assessment of the change in environmental enteropathy after supplementation with bovine lactoferrin and recombinant human lysozyme

Dr. Chrissie Thakwalakwa Telephone [redacted]

Your child is invited to participate in a research study conducted by Dr. Thakwalakwa and colleagues.

1. The purpose of this research is to determine whether giving a supplement of two different proteins will improve food absorption. The intervention will be powder of lactoferrin, a protein from cow's milk, and lysozyme, a human protein grown in rice, to try to help the intestine heal. These are the similar proteins to those found in breast milk. Each of these interventions has been separately shown to help improve intestinal function. Some children will receive these interventions but other children will receive interventions that look and taste just like these interventions but do not have any activity.

2. If you agree to enroll in the study, your child's participation is expected to last sixteen weeks or approximately four months. On the first day you will be asked some questions about your child's health and the health of your family, and your child will be measured and checked for swelling. Your child will have his/ her urine tested. The urine test consists of the following: 1. Your child will be asked to urinate into a plastic cup, and give the urine to the study team. 2. Your child will then be given a drink containing only sugar and water. 3. For 3 hours after the drink all urine will be collected in another plastic cup. 4. Your child will not be able to eat food for 1 hour after taking the drink of sugar water. Your child will also have a stool sample taken. This involves putting a diaper on the child, feeding the child, and collecting the stool after the child has produced stool.

You will return to the village healthcare site frequently to receive more supplement, to answer questions about if you child has had diarrhea, cough or fever, and to discuss your experience feeding the proteins to your child. Every 28 days after starting the study, you will bring your child back to have them measured. At 56 days and 112 days your child will have urine and stool collected for testing just like on the first day.

3. There is no cost to you for being in the study. The research project will provide all medicines, tests and medical care free to you.

4. There is a very small possibility of an allergic reaction to the supplements. If this occurs, your child will be taken out of the study. In addition, there is always the risk of developing previously

unknown side effects. The investigator is willing to discuss any questions you might have about these risks and discomforts.

5. There are no specific benefits to your child. Doctors will use the information they collect to understand how to heal the intestine of Malawian children.

6. Other than non-participation in the research, there are no alternatives.

7. All reasonable measures to protect the confidentiality of your child's records and your child's identity will be taken. There is a possibility that your child's medical record, including identifying information, may be inspected and photocopied by government or University officials or members of the Human Studies Committee.

8. If you have any questions or concerns regarding this study, if any problems arise, or there any feelings of pressure to participate, you may call an Investigator, Chrissie Thakwalakwa at [redacted] at the College of Medicine. You may also ask questions or state concerns regarding your (your child's) rights as a research subject to the COMREC Office at 011871911 at the College of Medicine.

9. In the event that your child experiences any complications due to participation in the study, University of Malawi investigators and their staffs will try to reduce, control, and treat any complications. If you feel that you are injured because of the study, please contact Chrissie Thakwalakwa ([redacted]) and/or the COMREC Office (011871911).

10. You (your child) will be informed of any significant new findings developed during the course of participation in this research that may have a bearing on your (your child's) willingness to continue in the study. The investigator may withdraw you (your child) from this research if circumstances arise (such as non-compliance with the protocol and non-tolerance of a study medication) which warrant doing so.

11. This research is not intended for the purpose of diagnosing or treating any medical problems not specifically stated in the purpose of the research.

I have read this consent form and have been given the opportunity to ask questions. I will also be given a copy of this consent form for my records. I hereby give my permission (or give permission for my child) to participate in the research described above.

Parent ___________________________
Name of child ____________________
Witness __________________________
